# Smoking Status, Including Non-Combustible Nicotine or Tobacco Products Use, and Site-Specific Colorectal Cancer Risk: A Nationwide Cohort Study

**DOI:** 10.3390/cancers18183010

**Published:** 2026-09-16

**Authors:** Seonghye Kim, Tae-Se Kim, Dong Wook Shin, Kyungdo Han, Byung-Hoon Min

**Affiliations:** 1 Department of Family Medicine & Supportive Care Center, Samsung Medical Center, Sungkyunkwan University School of Medicine, 50 Irwon-dong, Gangnam-gu, Seoul 06351, Republic of Korea; 2 Department of Medicine, Samsung Medical Center, Sungkyunkwan University School of Medicine, 50 Irwon-dong, Gangnam-gu, Seoul 06351, Republic of Korea; taese.kim@samsung.com; 3 Department of Clinical Research Design and Evaluation, Samsung Advanced Institute for Health Science and Technology (SAIHST), Sungkyunkwan University, 50 Irwon-dong, Gangnam-gu, Seoul 06351, Republic of Korea; 4 Department of Digital Health, Samsung Advanced Institute for Health Science and Technology (SAIHST), Sungkyunkwan University, 50 Irwon-dong, Gangnam-gu, Seoul 06351, Republic of Korea; 5 Department of Statistics and Actuarial Science, Soongsil University, 369 Sangdo-ro, Dongjak-gu, Seoul 06978, Republic of Korea

**Keywords:** colorectal cancer, smoking, combustible cigarette, non-combustible nicotine or tobacco product

## Abstract

Purpose: Cigarette smoking is an established risk factor for colorectal cancer (CRC), yet the association between smoking status, including non-combustible nicotine or tobacco product (NNTP) use, and site-specific CRC risk remains unclear. Methods: This population-based retrospective cohort study used data from 4,489,451 participants in the Korean National Health Insurance System database. Smoking status was classified according to combustible cigarette (CC) and NNTP use. Multivariable Cox regression models were used to estimate adjusted hazard ratios (aHRs) for CRC risk. Results: During a mean follow-up of 3.4 ± 0.4 years, 17,538 incident CRC cases occurred. Compared with never smoking, exclusive CC use was associated with the highest CRC risk (aHR 1.29, 95% CI 1.23–1.35), with elevated risks across the colon, rectum, distal colon, and unspecified sites. Rectal cancer risk was higher for switching from CCs to NNTPs (aHR 1.51, 95% CI 1.19–1.92) and concurrent CC and NNTP use (aHR 1.23, 95% CI 1.04–1.45). Compared with exclusive CC use, all other smoking-status groups had lower CRC risks, with subsite variations. Colon cancer risk was lower for switching from CCs to NNTPs and concurrent CC and NNTP use; rectal cancer risk was lowest for exclusive NNTP use; and distal colon cancer risk was lowest for concurrent CC and NNTP use. Stratified analyses showed significant interactions of smoking status with age and alcohol consumption in relation to CRC incidence. Conclusions: CRC risk varied by smoking status and anatomical subsite. Further studies are needed to clarify the long-term effects of NNTP use on CRC risk.

## Simple Summary

As smokers increasingly switch to or combine combustible cigarettes (CCs) with non-combustible nicotine or tobacco products (NNTPs), including liquid-type e-cigarettes and heated tobacco products, how smoking status and changes in use patterns relate to colorectal cancer risk remains unclear. In this nationwide Korean cohort of over 4.4 million adults, compared with never smoking, exclusive CC use was associated with the highest overall colorectal cancer risk, with elevated risk across the colon, rectum, and distal colon, but not proximal colon; rectal cancer risk was also higher among those who switched to or concurrently used NNTPs. Compared with exclusive CC use, however, all other smoking-status groups, including never smoking, former smoking, exclusive NNTP use, switching to NNTPs, and concurrent use, had lower colorectal cancer risk, though the magnitude varied by subsite. These findings indicate that the association between smoking status and colorectal cancer risk depends on both the comparison group and cancer subsite, underscoring the need for long-term studies to clarify the effects of NNTP use on colorectal cancer risk.

## 1. Introduction

Cigarette smoking is a well-established risk factor for colorectal cancer (CRC) [1]. Cigarette smoke contains over 9000 chemicals, including numerous established carcinogens such as tobacco-specific nitrosamines and polycyclic aromatic hydrocarbons [2]. Carcinogens from smoking reach the colorectal mucosa either directly through ingestion or via systemic circulation, exerting oncogenic effects on both the colon and rectum [3]. Against this background, a non-combustible nicotine or tobacco products (NNTPs), which deliver nicotine without tobacco combustion, have been promoted as a potentially less harmful alternative and are increasingly used worldwide, particularly for smoking cessation or harm reduction [4]. However, NNTP aerosols contain nicotine, thermal degradation products of propylene glycol, and flavoring agents that can induce oxidative stress, inflammation, apoptosis, DNA damage, and genotoxicity, processes that have been linked to tumor promotion and carcinogenesis [5,6]. Consequently, it remains uncertain whether switching from combustible cigarettes (CCs) to NNTPs confers net health benefits or introduces new long-term health risks.

CRC is biologically heterogeneous, with site-specific susceptibility to environmental exposures [7,8,9,10]. Previous studies have reported inconsistent findings regarding the association between smoking and CRC subsites, with strongest association observed for rectal cancer [8], right-sided colon cancer [9], or left-sided colon cancer [10] depending on the study. However, population-based evidence on CRC risk according to detailed categories of CC and NNTP use, including potential differences by CRC subsite, remains scarce [6].

Therefore, using a large nationwide cohort, we aimed to investigate the associations of smoking, including CC and NNTP use, and changes in use patterns, with CRC risk across colorectal subsites.

## 2. Materials and Methods

### 2.1. Data Source

This population-based retrospective cohort study was conducted using a customized dataset released by the Korean National Health Insurance Service (KNHIS), constructed as a stratified random sample of 40% of the Korean population. The dataset links eligibility and enrollment records, reimbursement claims coded according to the International Classification of Diseases, Tenth Revision (ICD-10), anthropometric and laboratory measurements obtained at general health examinations, and self-reported lifestyle information [11,12,13]. Enrollment in the national insurance program is compulsory for residents of Korea; premium-paying enrollees account for the great majority of the population, while the remaining 3%, who are unable to afford premiums, receive care through the government-funded Medical Aid program [11,12,13]. A general health examination is offered every two years to premium-paying enrollees, whether employed or self-employed, irrespective of age, and to all residents aged 40 years or older [11,12,13]. Because this system captures virtually the entire population, datasets derived from it have been widely used as a nationally representative resource for epidemiologic research in Korea [13,14,15].

### 2.2. Study Population

**Figure 1:**
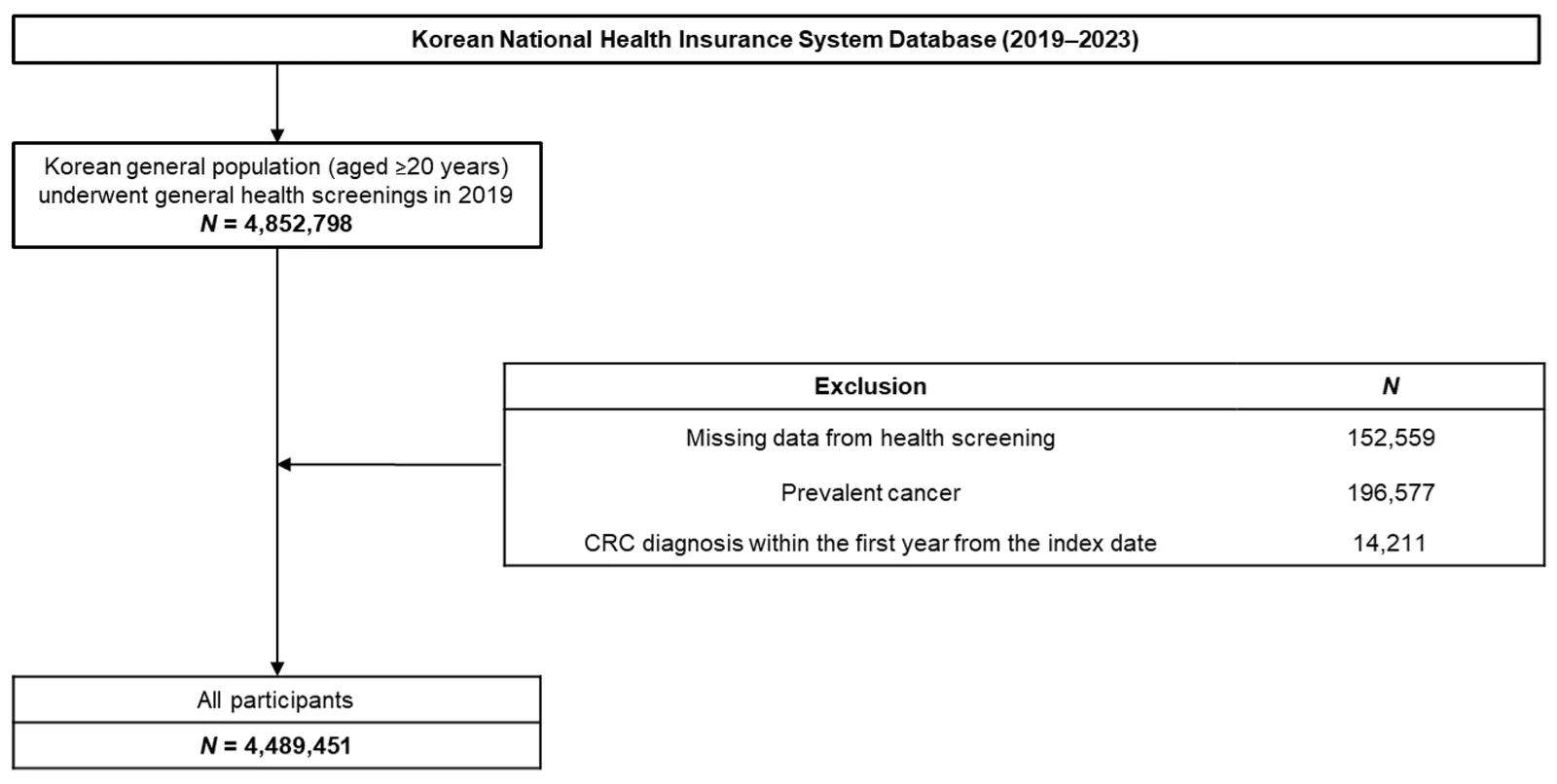
Flowchart of study participant enrollment (all participants). Abbreviations: CRC, colorectal cancer; N, number.

Initially, 4,852,798 individuals aged 20 years or older, who participated in the general health screenings in 2019 were included. This approach enabled us to assess smoking status and changes in use patterns. Participants who (1) had missing data from health screening (*n* = 152,559), (2) had a prevalent cancer diagnosis (*n* = 196,577), or (3) were diagnosed with CRC within the first year from the index date (*n* = 14,211) were excluded. The third criterion applied a one-year lag period to reduce the possibility of reverse causation, in which undetected preclinical disease could have prompted the changes in smoking behavior recorded at baseline. Finally, 4,489,451 participants were included in this study (Figure 1).

### 2.3. Ethical Statement

This study was approved by the Institutional Review Board of Soongsil university (IRB file no. SSU-202409-HR-581-1). Only de-identified data under confidentiality guidelines were used, and the requirement for informed consent was waived.

### 2.4. Smoking Status and Changes in Use Patterns

In this study, smoking status was classified into six categories: never smoking, former smoking, exclusive NNTP use, switching from CCs to NNTPs, concurrent CC and NNTP use, and exclusive CC use. Smoking status and changes in use patterns were assessed using self-administered questionnaires completed during the 2019 general health screenings.

Participants who reported having smoked ≥100 cigarettes in their lifetime were classified as people who currently or formerly smoked according to their current smoking status; those who reported having smoked <100 cigarettes were classified as people who had never smoked [16]. Former smoking comprised participants with a lifetime history of ≥100 CC who reported no current CC or NNTP use, and therefore included only individuals with prior CC use.

Current NNTP use was defined as answering “Yes” to either of the following questions: “In the past 30 days, have you used nicotine-containing liquid-type e-cigarettes?” or “Do you currently smoke heated tobacco products (heated cigarettes)?” [17]. Exclusive NNTP use was defined as current NNTP use among participants who had never smoked. Switching from CCs to NNTPs was defined as former CC use with current NNTP use. Concurrent CC and NNTP use was defined as current use of both products. Smoking status and product use were ascertained as reported at the 2019 general health screening, which served as the reference point for cessation and changes in use patterns; the questionnaire did not record when these changes occurred.

### 2.5. Study Outcomes and Follow-Up

We identified incident CRC using an operational definition that incorporated both ICD-10 codes (C18–20) and the Rare and Intractable Disease registration codes (V193), which provides greater accuracy than using only the ICD-10 code(s) for the primary diagnosis [18]. This definition has been previously validated, showing sensitivity and positive predictive values exceeding 90% [18,19]. We further investigated CRC subsites, including colon cancer (C18–19), proximal colon cancer (C18.0–18.4, comprising the cecum through the transverse colon), distal colon cancer (C18.5–18.7, C19, comprising the splenic flexure through the rectosigmoid junction), rectal cancer (C20), and unspecified colon cancer (C18.8–18.9, comprising overlapping lesions and colon cancer of unspecified site). Cases assigned to unspecified sites were retained and analyzed as a separate category rather than excluded; proximal and distal subsites therefore do not sum to total colon cancer. Follow-up for participants continued from the index date until the occurrence of a CRC, death, emigration, or 31 December 2023, whichever occurred first.

### 2.6. Covariates

Age was presented as a categorical variable with three groups: 20‒39 years, 40–64 years, and ≥65 years. Income levels were assessed according to monthly insurance premiums [20]. Comorbidities (hypertension [HTN], diabetes mellitus [DM], and dyslipidemia [DL]) were defined via corresponding ICD-10 codes and prescription claims [20]. Information on cigarettes per day, smoking duration, and pack-years for CC use was obtained [16]. Alcohol consumption was divided into two categories: non-drinker and drinker (drinking alcohol ≥ 2 times per month). Regular exercise was defined as engaging moderate-intensity activity for more than 30 min per session (≥5 days/week) or vigorous activity for more than 20 min per session (≥3 days/week). Body mass index (BMI) was calculated by dividing body weight in kilograms into the square of height in meters (kg/m^2^) [21].

### 2.7. Statistical Analysis

Descriptive statistics were performed using analysis of variance for continuous variables and the chi-squared test for categorical variables. Cox proportional hazards regression models were used to estimate hazard ratios (HRs) and 95% confidence intervals (CIs) for CRC risk according to smoking status and changes in use patterns. Models were adjusted for age, sex, income, BMI, comorbidities (HTN, DM, and DL), and health behaviors (alcohol consumption and regular exercise) as potential confounders. As a sensitivity analysis, CRC risk was examined among participants with a history of CC use, using exclusive CC use as the reference, with additional adjustment for cumulative CC use (pack-years). To account for multiple comparisons across colorectal subsites, the Benjamini–Hochberg procedure was applied to control the false discovery rate [22]. Stratification analyses were further conducted to explore potential effect modification by sex, age, and alcohol consumption.

All statistical analyses were conducted using SAS version 9.4 (SAS Institute Inc., Cary, NC, USA), and *p* < 0.05 was considered statistically significant.

## 3. Results

### 3.1. Baseline Characteristics of Participants

A total of 4,489,451 participants were included in the study. Regarding smoking status, participants who had never smoked comprised the largest group (*n* = 2,798,949), followed by those with exclusive CC use (*n* = 768,759), former smoking (*n* = 690,400), concurrent CC and NNTP use (*n* = 160,694), switching from CCs to NNTPs (*n* = 57,878), and exclusive NNTP use (*n* = 12,771). Compared with participants who had never smoked, those with exclusive NNTP use, switching from CCs to NNTPs, concurrent CC and NNTP use, exclusive CC use, or former smoking were more likely to be male, consume alcohol, and have a higher BMI. Participants with former smoking were more likely to be older and to have comorbidities than those in other groups (Table 1).

### 3.2. Risk of CRC by Smoking Status

During a mean follow-up period of 3.4 ± 0.4 years, 17,538 incident CRC cases occurred (Table 1).

Compared with participants who had never smoked, those with exclusive CC use had the highest CRC risk (adjusted HR [aHR] 1.29, 95% CI 1.23–1.35), followed by those with former smoking (aHR 1.10, 95% CI 1.05–1.16). Participants with exclusive NNTP use, those who switched from CCs to NNTPs, and those with concurrent CC and NNTP use showed no significant difference in CRC risk compared with participants who never smoked (Table 2).

Regarding CRC subsites, participants with exclusive CC use had a higher risk of colon cancer, rectal cancer, distal colon cancer, and CRC at unspecified sites than participants who had never smoked. The risk of rectal cancer was elevated among participants who switched from CCs to NNTPs (aHR 1.51, 95% CI 1.19–1.92) and those with concurrent CC and NNTP use (aHR 1.23, 95% CI 1.04–1.45) (Table 2).

Compared with participants with exclusive CC use, CRC risk was lower among those who had never smoked (aHR 0.78, 95% CI 0.74–0.82), those with former smoking (aHR 0.86, 95% CI 0.82–0.90), and those with concurrent CC and NNTP use (aHR 0.81, 95% CI 0.72–0.90). However, the risk reduction was not significant among participants with exclusive NNTP use or those who switched from CCs to NNTPs (Table 3).

When CRC subsites were further considered, risk estimates varied by smoking status. Compared with participants with exclusive CC use, colon cancer risk was lower among those who switched from CCs to NNTPs (aHR 0.68, 95% CI 0.53–0.87), those with concurrent CC and NNTP use (aHR 0.69, 95% CI 0.59–0.80), those who had never smoked (aHR 0.78, 95% CI 0.73–0.82), and those with former smoking (aHR 0.88, 95% CI 0.83–0.93). Rectal cancer risk was lower among participants with exclusive NNTP use (aHR 0.30, 95% CI 0.11–0.79), those who had never smoked (aHR 0.77, 95% CI 0.71–0.84), and those with former smoking (aHR 0.83, 95% CI 0.76–0.91). For distal colon cancer, the lowest risk was observed among participants with concurrent CC and NNTP use (aHR 0.60, 95% CI 0.44–0.82), followed by those who had never smoked (aHR 0.75, 95% CI 0.67–0.84) and those with former smoking (aHR 0.84, 95% CI 0.75–0.94). The risk of CRC at unspecified sites was also lower among participants who switched from CCs to NNTPs (aHR 0.64, 95% CI 0.46–0.88), those with concurrent CC and NNTP use (aHR 0.71, 95% CI 0.58–0.85), those who had never smoked (aHR 0.75, 95% CI 0.70–0.81), and those with former smoking (aHR 0.88, 95% CI 0.81–0.95) (Table 3).

These associations were materially unchanged after additional adjustment for cumulative CC use among participants with a history of CC use (Supplementary Table S1): concurrent CC and NNTP use remained significantly associated with lower risk of colorectal, colon, distal colon, and unspecified-site cancer compared with exclusive CC users (aHRs ranging from 0.57 to 0.81), and switching from CC to NNTP use remained significantly associated with lower risk of unspecified-site cancer (aHR 0.65, 95% CI 0.47–0.90), consistent with our main findings.

### 3.3. Risk of CRC by Stratified Analysis

**Figure 2:**
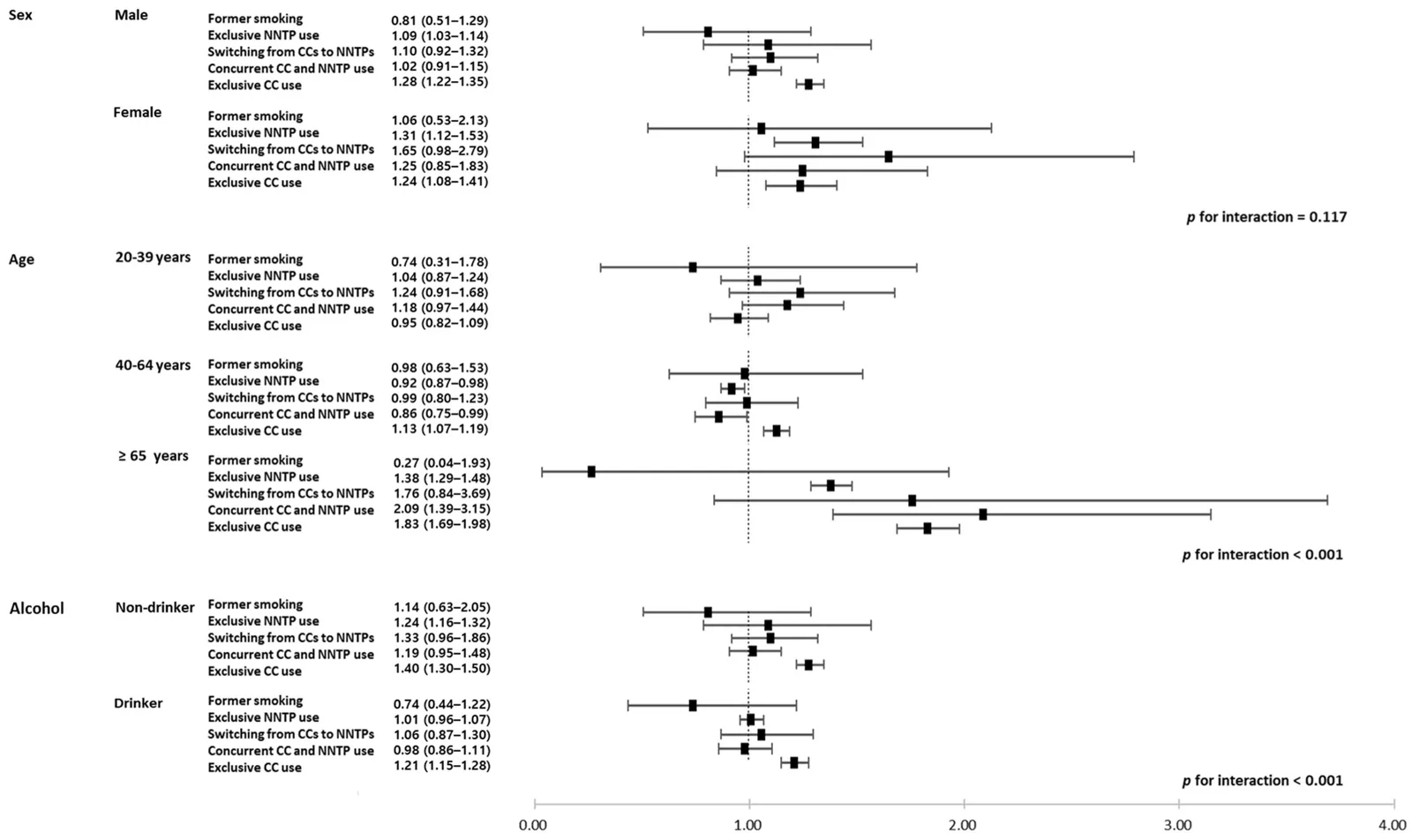
Stratified risk of colorectal cancer according to smoking status. Abbreviations: CC, combustible cigarette; CI, confidence interval; NNTP, noncombustible nicotine or tobacco product. Analyses were adjusted for age, sex, income (lowest quartile), body mass index, alcohol consumption, regular physical activity, and comorbidities (hypertension, diabetes mellitus, dyslipidemia). The horizontal dotted line represents the reference risk (adjusted HR = 1.00) for colorectal cancer among participants who had never smoked.

Stratified analyses showed significant interactions of smoking status with age (*p* for interaction < 0.001) and alcohol consumption (*p* for interaction < 0.001) in relation to CRC incidence. The association between smoking and CRC risk was more pronounced among participants aged ≥ 65 years and among those who did not consume alcohol (Figure 2).

## 4. Discussion

In this nationwide cohort study, site-specific CRC risk varied by smoking status. Exclusive CC use was associated with the highest CRC risk, whereas switching from CCs to NNTPs and concurrent CC and NNTP use were associated with heterogeneous risk patterns that differed across CRC subsites.

People who smoke CCs may switch to NNTPs or use both products concurrently because of the perception that NNTPs are less harmful than CCs. In our study, although switching from CCs to NNTPs and concurrent CC and NNTP use showed similar overall risk patterns, clear site-specific differences were observed. Compared with exclusive CC use, exclusive NNTP use was associated with a 70% lower rectal cancer risk, although the small number of events precludes firm conclusions. In contrast, no comparable reduction was observed among participants who switched from CCs to NNTPs or those with concurrent CC and NNTP use, suggesting that prior CC exposure may confer a persistent rectal cancer risk that is not fully mitigated by NNTP use. This pattern is consistent with previous studies reporting a stronger association between CC smoking and rectal cancer than for colon cancer [8,23]. Compared with exclusive CC use, switching from CCs to NNTPs and concurrent CC and NNTP use were associated with 32% and 31% lower risks of colon cancer, respectively. In analyses by anatomical subsite, concurrent CC and NNTP use was associated with a 40% lower risk of distal colon cancer, whereas no clear association was observed for proximal colon cancer across NNTP-related use patterns.

Overall, the lack of apparent risk reduction in rectal and proximal colon cancer among participants who switched from CCs to NNTPs and those with concurrent CC and NTTP use may suggest that the biological mechanisms underlying CC-related carcinogenesis differ across colorectal subsites. Prior studies have suggested that CC smoking may preferentially promote colorectal carcinogenesis through CpG island methylator phenotype (CIMP)-high or microsatellite instability (MSI)-high pathways, which are more characteristic of proximal colon cancer [24,25]. In this context, switching to or concurrently using NNTPs after prior CC exposure may not fully reverse these CC-related molecular alterations, potentially explaining the limited risk reduction observed for proximal colon cancer. This interpretation is further supported by the absence of a significant risk reduction for this subsite among participants with former smoking (Table 3; aHR 0.94, 95% CI 0.81–1.10, compared with exclusive CC use). In contrast, distal colon cancer is more frequently associated with chromosomal instability and TP53-driven pathways [26,27], which may differ in their susceptibility to CC-related carcinogenesis. This molecular heterogeneity may partly explain why switching from CCs to NNTPs and concurrent CC and NNTP use showed associations with lower distal colon cancer risk. The absence of a comparable reduction in rectal cancer risk may reflect persistent biological effects of prior CC exposure, potentially compounded by continued exposure to harmful constituents of NNTP aerosols [28,29,30]. However, these explanations remain speculative and warrant further mechanistic and longitudinal investigation.

In the present study, former smoking showed a more consistent reduction in CRC risk across subsites than exclusive CC use, with a significantly lower risk of overall CRC (aHR 0.86, 95% CI 0.82–0.90). This reduction extended to colon, distal colon, and rectal cancer, suggesting a broad benefit across anatomical sites. However, no significant risk reduction was observed for proximal colon cancer among participants with former smoking. These findings suggest that complete smoking cessation may confer broader CRC risk reduction than switching from CCs to NNTPs or concurrent CC and NNTP use, although the magnitude of benefit may vary across colorectal subsites.

We also evaluated CRC risk associated with exclusive NNTP use, which allowed us to explore the potential effects of NNTP use without prior CC exposure. Previous murine models have shown that NNTP exposure increases adenoma formation compared with air-exposed controls, although the magnitude of tumorigenesis appears weaker than that observed with CC exposure [31]. In humans, however, a prior systematic review reported that NNTP use was associated with elevations in cancer-related biomarkers, but that evidence directly linking these biomarker changes to increased cancer incidence remained inconclusive [6]. Consistent with this uncertainty, in our cohort, the estimate for exclusive NNTP use was based on a small number of events, and its confidence interval was compatible with both a reduction and an increase in overall CRC risk; firm conclusions regarding the risk associated with NNTP use in the absence of prior CC exposure cannot be drawn from these data. Nonetheless, a lower risk was observed among participants aged 40–64 years, but this subgroup estimate should be regarded as exploratory. Therefore, cohorts with larger numbers of events will be needed to determine whether risk differs across population subgroups.

These findings have implications for clinical counseling and tobacco policy. Although NNTPs are often promoted and perceived as harm-reduction alternatives to CCs [4], switching to or concurrently using NNTPs was not associated with a consistent reduction in CRC risk in this cohort, particularly for rectal and proximal colon cancer. By contrast, former smoking was associated with more consistent risk reductions across colorectal subsites, indicating that complete cessation of CC exposure, rather than substitution alone, remains central to CRC prevention. These observations do not support the positioning of NNTPs as a harm-reduction strategy for CRC prevention and underscore the need for cautious counseling of people who smoke CCs and are considering NNTPs. Public health messaging and regulatory decisions may benefit from accounting for site-specific CRC risks and from continued monitoring of evolving use patterns.

Several limitations of this study should be acknowledged. First, the follow-up duration was relatively short, because NNTP-use items were introduced into the screening questionnaire in 2019, and outcome data were available through 2023. This may have limited our ability to capture long-term colorectal carcinogenic effects, particularly for cancers with long latency periods. Second, smoking status was assessed at baseline using self-reported questionnaires, and changes in use patterns during follow-up were not captured. Because follow-up was relatively short, the extent of behavioral change after baseline is likely to have been smaller than in studies with longer observation periods. Some participants may nonetheless have changed their smoking behavior, and our estimates may be attenuated toward the null as a result. Studies with repeated assessment of tobacco use and longer follow-up are needed to determine how transitions between CC and NNTPs affect CRC risk. Third, although we adjusted for multiple demographic, clinical, and lifestyle factors, residual confounding cannot be excluded. Information on several established CRC risk factors was not available, including CRC screening practices (colonoscopy, fecal occult blood testing), diet (red and processed meat, fiber, calcium intake), family history of CRC, and medication use (aspirin, non-steroidal anti-inflammatory drugs), and the direction of any resulting bias cannot be determined. Studies incorporating these variables will be needed to address these sources of confounding. Fourth, detailed information on the intensity and cumulative exposure of tobacco use was unavailable. For NNTPs, and particularly for liquid-type e-cigarettes, the health screening questionnaire records use only as a binary response within the preceding 30 days, without information on frequency, quantity, or duration. For CCs, data such as age at initiation and time since cessation were not available in the dataset obtained for this analysis. These limitations precluded dose–response analyses and limited inferences regarding product-specific effects. Studies incorporating detailed exposure histories for both product types will be required to determine whether higher exposure is associated with increased CRC risk. Fifth, the number of events among participants with exclusive NNTP use was small, resulting in wide confidence intervals and limited statistical precision for this subgroup. Estimates for this group should therefore be interpreted with caution and require confirmation in cohorts with larger numbers of events. Sixth, deaths during follow-up were censored rather than analyzed using a subdistribution hazard approach, precluding absolute risk estimation in the presence of competing events. Finally, as our study was conducted in a Korean population, the findings may not be generalizable to other countries, where NNTP types, use patterns, and CRC epidemiology may differ. In particular, heated tobacco products account for the majority of NNTP use in Korea, whereas liquid-type e-cigarettes predominate in Western populations. Our findings should therefore not be extended to settings with different patterns of product use, and any implications for tobacco policy should be considered within the Korean context. Despite these limitations, the large nationwide cohort, detailed exposure classification, and subsite-specific analyses provide real-world evidence on the heterogeneous associations of CC and NNTP use with CRC risk.

## 5. Conclusions

In conclusion, during a mean follow-up of 3.4 ± 0.4 years, CRC risk varied by both smoking status and CRC subsite. Switching from CCs to NNTPs and concurrent CC and NNTP use were not uniformly associated with reduced CRC risk, particularly for rectal and proximal colon cancer. By contrast, complete smoking cessation was associated with more consistent risk reductions across subsites. These findings highlight the avoidance of CC exposure as the primary strategy for CRC prevention and underscore the need for cautious interpretation of harm-reduction approaches involving NNTPs.

## Figures and Tables

**Table 1:** Baseline characteristics of study participants.

Variables	Total Participants	Never Smoking	Former Smoking	Exclusive NNTP Use	Switching from CCs to NNTPs	Concurrent CC and NNTP Use	Exclusive CC Use	*p*-Values
	*n* = 4,489,451	*n* = 2,798,949	*n* = 690,400	*n* = 12,771	*n* = 57,878	*n* = 160,694	*n* = 768,759	
Age, years	49.0 ± 14.6	49.5 ± 15.4	52.6 ± 13.0	40.4 ± 12.1	39.4 ± 9.2	39.2 ± 9.7	46.7 ± 12.5	<0.001
20–39	1,247,880 (27.8)	789,973 (28.2)	115,450 (16.7)	6294 (49.3)	30,349 (52.4)	83,773 (52.1)	222,041 (28.9)	<0.001
40–64	2,596,649 (57.8)	1,543,817 (55.2)	452,789 (65.6)	6028 (47.2)	27,038 (46.7)	75,499 (47.0)	491,478 (63.9)	
≥65	644,922 (14.4)	465,159 (16.6)	122,161 (17.7)	449 (3.5)	491 (0.9)	1422 (0.9)	55,240 (7.2)	
Sex, male	2,349,838 (52.3)	801,595 (28.6)	641,162 (92.9)	9189 (72.0)	52,119 (90.1)	147,282 (91.7)	698,491 (90.9)	<0.001
Income (lowest 25%)	895,248 (19.9)	628,146 (22.4)	99,461 (14.4)	2194 (17.2)	5672 (9.8)	18,772 (11.7)	141,003 (18.3)	<0.001
CC smoking status								
Cigarettes/day	14.1 ± 7.6	N/A	14.7 ± 8.8	N/A	12.8 ± 6.1	13.0 ± 6.1	13.3 ± 6.5	
Smoking duration, years	18.3 ± 10.9	N/A	16.1 ± 10.2	N/A	14.9 ± 8.3	16.8 ± 8.7	20.0 ± 10.3	
Pack-years	14.1 ± 12.8	N/A	13.4 ± 13.6	N/A	10.4 ± 8.8	11.7 ± 9.4	14.9 ± 12.1	
Drinker	2,299,208 (51.2)	1,079,379 (38.6)	474,249 (68.7)	8945 (70.0)	43,878 (75.8)	124,933 (77.8)	567,824 (73.9)	<0.001
Regular exercise	1,297,512 (28.9)	803,498 (28.7)	232,796 (33.7)	3290 (25.8)	15,331 (26.5)	40,579 (25.3)	202,018 (26.3)	<0.001
Comorbidities								<0.001
HTN	1,286,491 (28.7)	754,790 (27.0)	265,414 (38.4)	2616 (20.5)	11,054 (19.1)	31,629 (19.7)	220,988 (28.8)	
DM	508,421 (11.3)	271,040 (9.7)	109,940 (15.9)	1119 (8.8)	4436 (7.7)	13,880 (8.6)	108,006 (14.1)	
DL	805,663 (18.0)	505,498 (18.1)	162,304 (23.5)	1328 (10.4)	5128 (8.9)	14,760 (9.2)	116,645 (15.2)	
BMI, kg/m^2^	24.3 ± 3.6	23.9 ± 3.6	25.0 ± 3.2	24.7 ± 3.9	25.3 ± 3.7	25.4 ± 3.8	24.7 ± 3.7	<0.001
Incident cancers by subsites								<0.001
Colorectum	17,538 (0.4)	10,378 (0.4)	3400 (0.5)	26 (0.2)	136 (0.2)	350 (0.2)	3248 (0.4)	
Colon	12,792 (0.3)	7870 (0.3)	2460 (0.4)	22 (0.2)	64 (0.1)	184 (0.1)	2192 (0.3)	
Rectum	4746 (0.1)	2508 (0.1)	940 (0.1)	4 (<0.1)	72 (0.1)	166 (0.1)	1056 (0.1)	
Proximal Colon	2017 (<0.1)	1311 (0.1)	387 (0.1)	4 (<0.1)	8 (<0.1)	23 (<0.1)	284 (<0.1)	
Distal Colon	2944 (0.1)	1641 (0.1)	649 (0.1)	6 (0.1)	18 (<0.1)	42 (<0.1)	588 (0.1)	
Unspecified	7831 (0.2)	4918 (0.2)	1424 (0.2)	12 (0.1)	38 (0.1)	119 (0.1)	1320 (0.2)	
Follow-up durations								
Mean ± sd, years	3.4 ± 0.4	3.4 ± 0.4	3.4 ± 0.4	3.3 ± 0.3	3.3 ± 0.3	3.4 ± 0.3	3.4 ± 0.4	<0.001
Median (Q1–Q3), years	3.4 (3.1–3.6)	3.4 (3.1–3.7)	3.4 (3.1–3.6)	3.3 (3.1–3.6)	3.3 (3.1–3.6)	3.3 (3.1–3.6)	3.3 (3.1–3.6)	<0.001

Data are expressed as the means ± standard deviation (sd) or number (%). BMI, body mass index; CC, combustible cigarette; DL, dyslipidemia; DM, diabetes mellitus; HTN, hypertension; NNTP, noncombustible nicotine or tobacco product; Q, quartile; sd, standard deviation.

**Table 2:** Subsite-specific risk of colorectal cancer according to smoking status: never smoking as reference.

Smoking Status	Participants (*n*)	Events (*n*)	Durations (PY)	IR (per 1000 PY)	Model 1 aHRs (95% CI)	Model 2 aHRs (95% CI)	FDR Adjusted *p*-Value
Colorectum							<0.001
Never smoking	2,798,949	10,378	9,480,954	1.09	1.00 (Reference)	1.00 (Reference)	
Former smoking	690,400	3400	2,332,302	1.46	1.33 (1.28, 1.39)	1.10 (1.05, 1.16)	
Exclusive NNTP use	12,771	26	42,626	0.61	0.56 (0.38, 0.82)	0.88 (0.60, 1.29)	
Switching from CCs to NNTPs	57,878	136	193,363	0.70	0.65 (0.54, 0.76)	1.14 (0.96, 1.36)	
Concurrent CC and NNTP use	160,694	350	538,662	0.65	0.60 (0.54, 0.66)	1.04 (0.93, 1.16)	
Exclusive CC use	768,759	3248	2,572,254	1.26	1.16 (1.11, 1.20)	1.29 (1.23, 1.35)	
Colon							<0.001
Never smoking	2,798,949	7870	9,480,954	0.83	1.00 (Reference)	1.00 (Reference)	
Former smoking	690,400	2460	2,332,302	1.05	1.27 (1.22, 1.33)	1.13 (1.07, 1.19)	
Exclusive NNTP use	12,771	22	42,626	0.52	0.62 (0.41, 0.95)	1.14 (0.75, 1.73)	
Switching from CCs to NNTPs	57,878	64	193,363	0.33	0.40 (0.31, 0.51)	0.88 (0.68, 1.12)	
Concurrent CC and NNTP use	160,694	184	538,662	0.34	0.41 (0.36, 0.48)	0.89 (0.76, 1.03)	
Exclusive CC use	768,759	2192	2,572,254	0.85	1.03 (0.98, 1.08)	1.29 (1.22, 1.36)	
Rectum							<0.001
Never smoking	2,798,949	2508	9,480,954	0.26	1.00 (Reference)	1.00 (Reference)	
Former smoking	690,400	940	2,332,302	0.40	1.52 (1.41, 1.64)	1.08 (0.99, 1.18)	
Exclusive NNTP use	12,771	4	42,626	0.09	0.36 (0.13, 0.95)	0.38 (0.14, 1.02)	
Switching from CCs to NNTPs	57,878	72	193,363	0.37	1.41 (1.12, 1.79)	1.51 (1.19, 1.92)	
Concurrent CC and NNTP use	160,694	166	538,662	0.31	1.17 (0.99, 1.37)	1.23 (1.04, 1.45)	
Exclusive CC use	768,759	1056	2,572,254	0.41	1.56 (1.45, 1.67)	1.30 (1.19, 1.42)	
Proximal colon							0.020
Never smoking	2,798,949	1311	9,480,954	0.14	1.00 (Reference)	1.00 (Reference)	
Former smoking	690,400	387	2,332,302	0.17	1.20 (1.07, 1.34)	1.03 (0.90, 1.18)	
Exclusive NNTP use	12,771	4	42,626	0.09	0.68 (0.25, 1.81)	1.49 (0.56, 3.97)	
Switching from CCs to NNTPs	57,878	8	193,363	0.04	0.30 (0.15, 0.60)	0.87 (0.43, 1.75)	
Concurrent CC and NNTP use	160,694	23	538,662	0.04	0.31 (0.20, 0.47)	0.88 (0.57, 1.34)	
Exclusive CC use	768,759	284	2,572,254	0.11	0.80 (0.70, 0.91)	1.09 (0.94, 1.27)	
Distal colon							<0.001
Never smoking	2,798,949	1641	9,480,954	0.17	1.00 (Reference)	1.00 (Reference)	
Former smoking	690,400	649	2,332,302	0.28	1.61 (1.47, 1.76)	1.11 (0.99, 1.24)	
Exclusive NNTP use	12,771	6	42,626	0.14	0.81 (0.37, 1.82)	1.29 (0.58, 2.88)	
Switching from CCs to NNTPs	57,878	18	193,363	0.09	0.54 (0.34, 0.86)	0.98 (0.61, 1.56)	
Concurrent CC and NNTP use	160,694	42	538,662	0.08	0.45 (0.33, 0.61)	0.80 (0.58, 1.10)	
Exclusive CC use	768,759	588	2,572,254	0.23	1.32 (1.20, 1.45)	1.33 (1.19, 1.49)	
Unspecified							<0.001
Never smoking	2,798,949	4918	9,480,954	0.52	1.00 (Reference)	1.00 (Reference)	
Former smoking	690,400	1424	2,332,302	0.61	1.18 (1.11, 1.25)	1.16 (1.08, 1.25)	
Exclusive NNTP use	12,771	12	42,626	0.28	0.55 (0.31, 0.96)	1.00 (0.57, 1.76)	
Switching from CCs to NNTPs	57,878	38	193,363	0.20	0.38 (0.28, 0.53)	0.84 (0.61, 1.17)	
Concurrent CC and NNTP use	160,694	119	538,662	0.22	0.43 (0.36, 0.51)	0.94 (0.78, 1.13)	
Exclusive CC use	768,759	1320	2,572,254	0.51	0.99 (0.93, 1.06)	1.33 (1.23, 1.43)	

aHRs, adjusted hazard ratios; CI, confidence interval; CC, combustible cigarette; FDR, false discovery rate; n, number; IR, incidence rates; NNTP, noncombustible nicotine or tobacco product; PY, person-years. Model 1: unadjusted; Model 2: adjusted for age, sex, income (lowest quartile), body mass index, alcohol consumption, regular exercise, and comorbidities (hypertension, diabetes mellitus, dyslipidemia).

**Table 3:** Subsite-specific risk of colorectal cancer according to smoking status: exclusive combustible cigarette use as reference.

Smoking Status	Participants (*n*)	Events (*n*)	Durations (PY)	Incidence Rates (per 1000 PY)	Model 1 aHRs (95% CI)	Model 2 aHRs (95% CI)
Colorectum						
Exclusive CC use	768,759	3248	2,572,254	1.26	1.00 (Reference)	1.00 (Reference)
Former smoking	690,400	3400	2,332,302	1.46	1.15 (1.10, 1.21)	0.86 (0.82, 0.90)
Exclusive NNTP use	12,771	26	42,626	0.61	0.48 (0.33, 0.71)	0.68 (0.46, 1.01)
Switching from CCs to NNTPs	57,878	136	193,363	0.70	0.56 (0.47, 0.66)	0.89 (0.75, 1.06)
Concurrent CC and NNTP use	160,694	350	538,662	0.65	0.52 (0.46, 0.58)	0.81 (0.72, 0.90)
Never smoking	2,798,949	10,378	9,480,954	1.09	0.87 (0.83, 0.90)	0.78 (0.74, 0.82)
Colon						
Exclusive CC use	768,759	2192	2,572,254	0.85	1.00 (Reference)	1.00 (Reference)
Former smoking	690,400	2460	2,332,302	1.05	1.24 (1.17, 1.31)	0.88 (0.83, 0.93)
Exclusive NNTP use	12,771	22	42,626	0.52	0.61 (0.40, 0.92)	0.88 (0.58, 1.34)
Switching from CCs to NNTPs	57,878	64	193,363	0.33	0.39 (0.30, 0.50)	0.68 (0.53, 0.87)
Concurrent CC and NNTP use	160,694	184	538,662	0.34	0.40 (0.35, 0.47)	0.69 (0.59, 0.80)
Never smoking	2,798,949	7870	9,480,954	0.83	0.97 (0.93, 1.02)	0.78 (0.73, 0.82)
Rectum						
Exclusive CC use	768,759	1056	2,572,254	0.41	1.00 (Reference)	1.00 (Reference)
Former smoking	690,400	940	2,332,302	0.40	0.98 (0.90, 1.07)	0.83 (0.76, 0.91)
Exclusive NNTP use	12,771	4	42,626	0.09	0.23 (0.09, 0.61)	0.30 (0.11, 0.79)
Switching from CCs to NNTPs	57,878	72	193,363	0.37	0.91 (0.72, 1.15)	1.16 (0.91, 1.48)
Concurrent CC and NNTP use	160,694	166	538,662	0.31	0.75 (0.64, 0.88)	0.95 (0.80, 1.12)
Never smoking	2,798,949	2508	9,480,954	0.26	0.64 (0.60, 0.69)	0.77 (0.71, 0.84)
Proximal colon						
Exclusive CC use	768,759	284	2,572,254	0.11	1.00 (Reference)	1.00 (Reference)
Former smoking	690,400	387	2,332,302	0.17	1.50 (1.29, 1.75)	0.94 (0.81, 1.10)
Exclusive NNTP use	12,771	4	42,626	0.09	0.85 (0.32, 2.28)	1.36 (0.51, 3.64)
Switching from CCs to NNTPs	57,878	8	193,363	0.04	0.37 (0.19, 0.76)	0.79 (0.39, 1.60)
Concurrent CC and NNTP use	160,694	23	538,662	0.04	0.39 (0.25, 0.59)	0.80 (0.52, 1.23)
Never smoking	2,798,949	1311	9,480,954	0.14	1.25 (1.10, 1.42)	0.91 (0.79, 1.06)
Distal colon						
Exclusive CC use	768,759	588	2,572,254	0.23	1.00 (Reference)	1.00 (Reference)
Former smoking	690,400	649	2,332,302	0.28	1.22 (1.09, 1.36)	0.84 (0.75, 0.94)
Exclusive NNTP use	12,771	6	42,626	0.14	0.62 (0.28, 1.38)	0.97 (0.43, 2.17)
Switching from CCs to NNTPs	57,878	18	193,363	0.09	0.41 (0.26, 0.65)	0.73 (0.46, 1.17)
Concurrent CC and NNTP use	160,694	42	538,662	0.08	0.34 (0.25, 0.47)	0.60 (0.44, 0.82)
Never smoking	2,798,949	1641	9,480,954	0.17	0.76 (0.69, 0.83)	0.75 (0.67, 0.84)
Unspecified						
Exclusive CC use	768,759	1320	2,572,254	0.51	1.00 (Reference)	1.00 (Reference)
Former smoking	690,400	1424	2,332,302	0.61	1.19 (1.10, 1.28)	0.88 (0.81, 0.95)
Exclusive NNTP use	12,771	12	42,626	0.28	0.55 (0.31, 0.97)	0.75 (0.43, 1.33)
Switching from CCs to NNTPs	57,878	38	193,363	0.20	0.38 (0.28, 0.53)	0.64 (0.46, 0.88)
Concurrent CC and NNTP use	160,694	119	538,662	0.22	0.43 (0.36, 0.52)	0.71 (0.58, 0.85)
Never smoking	2,798,949	4918	9,480,954	0.52	1.01 (0.95, 1.07)	0.75 (0.70, 0.81)

aHRs, adjusted hazard ratios; CI, confidence interval; CC, combustible cigarette; n, number; NNTP, noncombustible nicotine or tobacco product; PY, person-years. Model 1: unadjusted; Model 2: adjusted for age, sex, income (lowest quartile), body mass index, alcohol consumption, regular exercise, and comorbidities (hypertension, diabetes mellitus, dyslipidemia).

## Data Availability

The data used in this study is not available to the public. They are available only to licensed researchers authorized to receive access by the KNHIS.

## Supplementary Materials

The following supporting information can be downloaded at: https://www.mdpi.com/article/10.3390/cancers18183010/s1, Table S1: Subsite-specific risk of colorectal cancer according to smoking status: exclusive combustible cigarette use as reference.

## Abbreviations

aHR	adjusted hazard ratio
BMI	body mass index
CC	combustible cigarette
CI	confidence interval
CRC	colorectal cancer
DM	diabetes mellitus
DL	dyslipidemia
HTN	hypertension
ICD-10	International Classification of Diseases Tenth Revision
IRB	institutional review board
KNHIS	Korean National Health Insurance System
NNTP	non-combustible nicotine or tobacco product
RID	rare and intractable diseases

## References

[1] Cheng J., Chen Y., Wang X., Wang J., Yan Z., Gong G., Li G., Li C. (2015). Meta-analysis of prospective cohort studies of cigarette smoking and the incidence of colon and rectal cancers. Eur. J. Cancer Prev..

[2] Li Y., Hecht S.S. (2022). Carcinogenic components of tobacco and tobacco smoke: A 2022 update. Food Chem. Toxicol..

[3] Gram I.T., Park S.Y., Wilkens L.R., Haiman C.A., Le Marchand L. (2020). Smoking-Related Risks of Colorectal Cancer by Anatomical Subsite and Sex. Am. J. Epidemiol..

[4] Erhabor J., Boakye E., Obisesan O., Osei A.D., Tasdighi E., Mirbolouk H., DeFilippis A.P., Stokes A.C., Hirsch G.A., Benjamin E.J. (2023). E-Cigarette Use Among US Adults in the 2021 Behavioral Risk Factor Surveillance System Survey. JAMA Netw. Open.

[5] Gordon T., Karey E., Rebuli M.E., Escobar Y.H., Jaspers I., Chen L.C. (2022). E-Cigarette Toxicology. Annu. Rev. Pharmacol. Toxicol..

[6] Kundu A., Sachdeva K., Feore A., Sanchez S., Sutton M., Seth S., Schwartz R., Chaiton M. (2025). Evidence update on the cancer risk of vaping e-cigarettes: A systematic review. Tob. Induc. Dis..

[7] Huyghe J.R., Harrison T.A., Bien S.A., Hampel H., Figueiredo J.C., Schmit S.L., Conti D.V., Chen S., Qu C., Lin Y. (2021). Genetic architectures of proximal and distal colorectal cancer are partly distinct. Gut.

[8] Botteri E., Iodice S., Bagnardi V., Raimondi S., Lowenfels A.B., Maisonneuve P. (2008). Smoking and colorectal cancer: A meta-analysis. JAMA.

[9] Leufkens A.M., Van Duijnhoven F.J., Siersema P.D., Boshuizen H.C., Vrieling A., Agudo A., Gram I.T., Weiderpass E., Dahm C., Overvad K. (2011). Cigarette smoking and colorectal cancer risk in the European Prospective Investigation into Cancer and Nutrition study. Clin. Gastroenterol. Hepatol..

[10] Yang L.P., Wang Z.X., Zhang R., Zhou N., Wang A.M., Liang W., Wang Z.Q., Luo H.Y., Wang F., Liu J.W. (2021). Association between cigarette smoking and colorectal cancer sidedness: A multi-center big-data platform-based analysis. J. Transl. Med..

[11] Shin D.W., Cho B., Guallar E. (2016). Korean national health insurance database. JAMA Intern. Med..

[12] Seong S.C., Kim Y.-Y., Park S.K., Khang Y.H., Kim H.C., Park J.H., Kang H.-J., Do C.-H., Song J.-S., Lee E.-J. (2017). Cohort profile: The national health insurance service-national health screening cohort (NHIS-HEALS) in Korea. BMJ Open.

[13] Lim S.J., Jang S.I. (2025). Leveraging National Health Insurance Service Data for Public Health Research in Korea: Structure, Applications, and Future Directions. J. Korean Med. Sci..

[14] Jeon K.H., Jung J., Cho M.H., Lee D., Han K., Cho I.Y., Shin D.W. (2025). RA and risk of colorectal cancer: A nationwide cohort study. Rheumatology.

[15] Park Y.-M.M., Amick B.C., McElfish P.A., Brown C.C., Schootman M., Narcisse M.-R., Lee S.-S., Choi Y.J., Han K. (2025). Income dynamics and risk of colorectal cancer in individuals with type 2 diabetes: A nationwide population-based cohort study. J. Epidemiol..

[16] Centers for Disease Control and Prevention, National Center for Health Statistics National Health Interview Survey: Adult Tobacco Use—Glossary. https://archive.cdc.gov/www_cdc_gov/nchs/nhis/tobacco/tobacco_glossary.htm.

[17] Kim S.H., Cho H.J. (2020). Prevalence and correlates of current use of heated tobacco products among a nationally representative sample of Korean adults: Results from a cross-sectional study. Tob. Induc. Dis..

[18] Yang M.S., Park M., Back J.H., Lee G.H., Shin J.H., Km K., Seo H.J., Kim Y.A. (2022). Validation of cancer diagnosis based on the national health insurance service database versus the national cancer registry database in Korea. Cancer Res. Treat. Off. J. Korean Cancer Assoc..

[19] Park H., Kim Y.R., Pyun Y., Joo H., Shin A. (2023). Operational definitions of colorectal cancer in the Korean National Health Insurance Database. J. Prev. Med. Public Health.

[20] Shin D.W., Cho J., Park J.H., Cho B. (2022). National General Health Screening Program in Korea: History, current status, and future direction. Precis. Future Med..

[21] World Health Organization (2000). The Asia-Pacific Perspective: Redefining Obesity and Its Treatment.

[22] Benjamini Y., Hochberg Y. (1995). Controlling the false discovery rate: A practical and powerful approach to multiple testing. J. R. Stat. Soc. Ser. B (Methodol.).

[23] Paskett E.D., Reeves K.W., Rohan T.E., Allison M.A., Williams C.D., Messina C.R., Whitlock E., Sato A., Hunt J.R. (2007). Association between cigarette smoking and colorectal cancer in the Women’s Health Initiative. J. Natl. Cancer Inst..

[24] Botteri E., Borroni E., Sloan E.K., Bagnardi V., Bosetti C., Peveri G., Santucci C., Specchia C., van den Brandt P., Gallus S. (2020). Smoking and Colorectal Cancer Risk, Overall and by Molecular Subtypes: A Meta-Analysis. Am. J. Gastroenterol..

[25] Amitay E.L., Carr P.R., Jansen L., Roth W., Alwers E., Herpel E., Kloor M., Bläker H., Chang-Claude J., Brenner H. (2020). Smoking, alcohol consumption and colorectal cancer risk by molecular pathological subtypes and pathways. Br. J. Cancer.

[26] Farooqi A.A., de la Roche M., Djamgoz M.B.A., Siddik Z.H. (2019). Overview of the oncogenic signaling pathways in colorectal cancer: Mechanistic insights. Semin. Cancer Biol..

[27] Jin M., Shang F., Wu J., Fan Q., Chen C., Fan J., Liu L., Nie X., Zhang T., Cai K. (2021). Tumor-Associated Microbiota in Proximal and Distal Colorectal Cancer and Their Relationships with Clinical Outcomes. Front. Microbiol..

[28] Barleben A., Mills S. (2010). Anorectal anatomy and physiology. Surg. Clin. North. Am..

[29] Bonfiglio R., Sisto R., Casciardi S., Palumbo V., Scioli M.P., Palumbo A., Trivigno D., Giacobbi E., Servadei F., Melino G. (2024). The impact of toxic metal bioaccumulation on colorectal cancer: Unravelling the unexplored connection. Sci. Total Environ..

[30] Ganapathy V., Manyanga J., Brame L., McGuire D., Sadhasivam B., Floyd E., Rubenstein D.A., Ramachandran I., Wagener T., Queimado L. (2017). Electronic cigarette aerosols suppress cellular antioxidant defenses and induce significant oxidative DNA damage. PLoS ONE.

[31] Sayed I.M., Chakraborty A., Inouye K., Dugan L., Tocci S., Advani I., Park K., Gaboyan S., Kasaraneni N., Ma B. (2025). E-cigarettes increase the risk of adenoma formation in murine colorectal cancer model. Arch. Toxicol..

