# Supplementary material for: Smoking Status, Including Non-Combustible Nicotine or Tobacco Products Use, and Site-Specific Colorectal Cancer Risk: A Nationwide Cohort Study
Source: Cancers (Basel). 2026 Sep 16;18(18):3010. doi: 10.3390/cancers18183010 (PMC13605874; doi:10.3390/cancers18183010)
Supplement: Supplementary file 1 [file cancers-18-03010-s001.zip › cancers-4527213-supplementary.pdf]

Supplementary Table S1. Subsite-specific risk of colorectal cancer according to smoking status: exclusive combustible cigarette use as reference

Smoking status	Participants (*n*)	Events (*n*)	Durations (PY)	Incidence rates (per 1,000 PY)	aHRs (95% CI)
Colorectum					
Exclusive CC use	768,759	3,248	2,572,254	1.26	1.00 (Reference)
Former smoking	690,400	3,400	2,332,302	1.46	0.88 (0.84,0.92)
Switching from CCs to NNTPs	57,878	136	193,363	0.7	0.91 (0.76,1.08)
Concurrent CC and NNTP use	160,694	350	538,662	0.65	0.81 (0.71,0.89)
Colon					
Exclusive CC use	768,759	2,192	2,572,254	0.85	1.00 (Reference)
Former smoking	690,400	2,460	2,332,302	1.05	0.90 (0.85,0.95)
Switching from CCs to NNTPs	57,878	64	193,363	0.33	0.69 (0.55,0.89)
Concurrent CC and NNTP use	160,694	184	538,662	0.34	0.68 (0.58,0.79)
Rectum					
Exclusive CC use	768,759	1,056	2,572,254	0.41	1.00 (Reference)
Former smoking	690,400	940	2,332,302	0.4	0.85 (0.77,0.93)
Switching from CCs to NNTPs	57,878	72	193,363	0.37	1.18 (0.93,1.51)
Concurrent CC and NNTP use	160,694	166	538,662	0.31	0.95 (0.80,1.11)
Proximal colon					
Exclusive CC use	768,759	284	2,572,254	0.11	1.00 (Reference)
Former smoking	690,400	387	2,332,302	0.17	0.94 (0.81,1.10)
Switching from CCs to NNTPs	57,878	8	193,363	0.04	0.80 (0.39,1.61)

Concurrent CC and NNTP use	160,694	23	538,662	0.04	0.80 (0.52,1.23)
Distal colon					
Exclusive CC use	768,759	588	2,572,254	0.23	1.00 (Reference)
Former smoking	690,400	649	2,332,302	0.28	0.86 (0.76,0.95)
Switching from CCs to NNTPs	57,878	18	193,363	0.09	0.74 (0.46,1.19)
Concurrent CC and NNTP use	160,694	42	538,662	0.08	0.57 (0.42,0.79)
Unspecified					
Exclusive CC use	768,759	1320	2,572,254	0.51	1.00 (Reference)
Former smoking	690,400	1,424	2,332,302	0.61	0.90 (0.83,0.98)
Switching from CCs to NNTPs	57,878	38	193,363	0.2	0.65 (0.47,0.90)
Concurrent CC and NNTP use	160,694	119	538,662	0.22	0.71 (0.57,0.85)

aHRs, adjusted hazard ratios; CI, confidence interval; CC, combustible cigarette; NNTP, noncombustible nicotine or tobacco product; PY, person-years.

Adjusted for age, sex, income (lowest quartile), body mass index, alcohol consumption, regular exercise, comorbidities (hypertension, diabetes mellitus, dyslipidemia), and cumulative CC use (pack-years).
